# Supplementary material for: End Sequence Analysis Toolkit (ESAT) expands the extractable information from single-cell RNA-seq data
Source: Genome Res. 2016 Oct;26(10):1397–410. doi: 10.1101/gr.207902.116 (PMC5052061; doi:10.1101/gr.207902.116)
Supplement: Supplemental Material [file supp_gr.207902.116_Supp_Materials.tar.gz › ESAT-esat_v0.1/doc/html/classbroad_1_1pda_1_1annotation_1_1_locus-members.html]

ESAT: Member List


ESAT


- Main Page
- Related Pages
- Packages
- Classes
- Files

- Class List
- Class Index
- Class Hierarchy
- Class Members

All Classes Namespaces Files Functions Variables Enumerator Pages

- broad
- pda
- annotation
- Locus

broad.pda.annotation.Locus Member List

This is the complete list of members for broad.pda.annotation.Locus, including all inherited members.

|  |  |  |
| --- | --- | --- |
| addIsoform(GeneWithIsoforms iso, String set) | broad.pda.annotation.Locus |  |
| distanceToOppositeStrand5primeNeighbor(BEDFileParser geneSet) | broad.pda.annotation.Locus |  |
| getAllIsoforms(String excludeSet) | broad.pda.annotation.Locus |  |
| getAllIsoforms() | broad.pda.annotation.Locus |  |
| getAllMerged() | broad.pda.annotation.Locus |  |
| getComaptibleBetweenAny2Sets(ArrayList< String > setLst, boolean isFully) | broad.pda.annotation.Locus |  |
| getComaptibleIsoforms(Gene g, boolean isFully) | broad.pda.annotation.Locus |  |
| getDistanceTo3primeNeighbor(BEDFileParser geneSet) | broad.pda.annotation.Locus |  |
| getDistanceTo5primeNeighbor(BEDFileParser geneSet) | broad.pda.annotation.Locus |  |
| getExonUnion() | broad.pda.annotation.Locus |  |
| getIsComaptibleBetweenAny2Sets(ArrayList< String > setLst, boolean isFully) | broad.pda.annotation.Locus |  |
| getIsComaptibleBetweenAny2Sets(ArrayList< String > set3Lst, BEDFileParser uniqIsoBed) | broad.pda.annotation.Locus |  |
| getIsComaptibleBetweenSubsets(ArrayList< String > set1Lst, ArrayList< String > set2Lst, boolean isFully) | broad.pda.annotation.Locus |  |
| getIsComaptibleBetweenSubsets(ArrayList< String > set1Lst, ArrayList< String > set2Lst, BEDFileParser uniqIsoBed) | broad.pda.annotation.Locus |  |
| getIsComaptibleWithRefPerSet() | broad.pda.annotation.Locus |  |
| getIsPartiallyComaptibleWithRefPerSet() | broad.pda.annotation.Locus |  |
| getLongestIntornChainIso(String refSet) | broad.pda.annotation.Locus |  |
| getMaxExonsCovered() | broad.pda.annotation.Locus |  |
| getMaxPctCovered() | broad.pda.annotation.Locus |  |
| getMaxPctGenomeCovered() | broad.pda.annotation.Locus |  |
| getMaxScoreAcrossAllIso() | broad.pda.annotation.Locus |  |
| getMaxScorePerSet() | broad.pda.annotation.Locus |  |
| getMerged() | broad.pda.annotation.Locus |  |
| getNumberOfIsoforms() | broad.pda.annotation.Locus |  |
| getNumIsoformsPerSet() | broad.pda.annotation.Locus |  |
| getReferenceTranscript() | broad.pda.annotation.Locus |  |
| getSetIsoforms(String setName) | broad.pda.annotation.Locus |  |
| getUnqComaptibleBetweenAny2Sets(ArrayList< String > setLst, Collection<?extends Gene > unqIsoSet, boolean isFully) | broad.pda.annotation.Locus |  |
| getUnqComaptibleBetweenSubsets(ArrayList< String > set1Lst, ArrayList< String > set2Lst, Collection< Gene > unqIsoSet, boolean isFully) | broad.pda.annotation.Locus |  |
| isFullyComaptible(Gene iso1, Gene iso2, boolean stringent) | broad.pda.annotation.Locus | static |
| isTerminiCoverageBias() | broad.pda.annotation.Locus |  |
| Locus(GeneWithIsoforms g) | broad.pda.annotation.Locus |  |
| numOfOverlapSets(ArrayList< String > setLst) | broad.pda.annotation.Locus |  |
| pctOverlapWithRef() | broad.pda.annotation.Locus |  |
| SelectLongestIntronChainCandidate(Collection< Gene > betweenSubsetsIsoSet) | broad.pda.annotation.Locus | static |
| selectRandIsoSubset(Integer maxIsoPerLoci) | broad.pda.annotation.Locus |  |
| updateIsoformsNameWithLocusName() | broad.pda.annotation.Locus |  |


---

Generated on Fri Aug 15 2014 15:10:58 for ESAT by  

 1.8.7
